# Spontaneous regression of a giant uterine leiomyoma after delivery: a case report and literature review

**DOI:** 10.1186/s12884-024-06324-2

**Published:** 2024-02-10

**Authors:** Lifang Zhu-ge, Qiaoli Bei, Weiping Pan, Xiaojun Ni

**Affiliations:** 1 Department of Gynaecology, Beilun District People’s Hospotal, 1288 Lusan East Road, Ningbo, Zhejiang Province 315800 China; 2 Department of Gynaecology and Obstetric, The Second Affiliated Hospital of Zhejiang University School of Medicine, Lanxi Hospital, Jinhua, China

**Keywords:** Postpartum, Pregnancy, Regression, Uterine leiomyomas

## Abstract

**Background:**

Uterine leiomyomas are hormone-dependent benign tumors and often begin to shrink after menopause due to the reduction in ovarian steroids. The influence of pregnancy on uterine leiomyomas size remains unclear. Here, we present a case of spontaneous regression of a giant uterine leiomyoma after delivery.

**Case presentation:**

A 40-year-old woman presented with multiple uterine leiomyomas, one of which is a giant uterine leiomyomas (approximately 8 cm in diameter) that gradually shrinked after delivery. At over two months postpartum, the large myometrial leiomyoma had transformed into a submucosal leiomyoma, and over 3 years postpartum, both the submucosal leiomyoma and multiple intramural leiomyomas completely regressed.

**Conclusion:**

Spontaneous regression of a giant uterine leiomyom is rare after delivery. Considering uterine leiomyoma regression until over 3 year postpartum,we need to observe the regression of uterine fibroid for a longer time postpartum in the absence of fibroid related complications. In addition, it will provide new insights for treatment options of uterine leiomyomas in the future.

## Background

Uterine leiomyomas are the most common benign uterine tumors that are hormone-dependent. Approximately 50-77% of women suffer from uterine leiomyomas [1]. Uterine leiomyomas gradually shrink in postmenopausal women due to hormonal changes, while they typically exhibit gradual growth during the reproductive years.The influence of pregnancy on the size of uterine leiomyomas is unclear. But most literatures suggest that pregnancy has no effect on the size of uterine leiomyomas, which may cause uterine fibroid enlargement during the first trimester and uterine fibroid shrinkage or stabilization during the second and third trimesters. During puerperium, the majority of studies reported a size reduction or no changes of Uterine leiomyomas [2, 3]. This is a rare case report of a woman with multiple uterine leiomyomas (the largest leiomyoma measuring > 8.0 cm) who experienced complete regression of the submucosal and multiple intramural leiomyomas more than 3 years postpartum.

## Case presentation

**Fig. 1:**
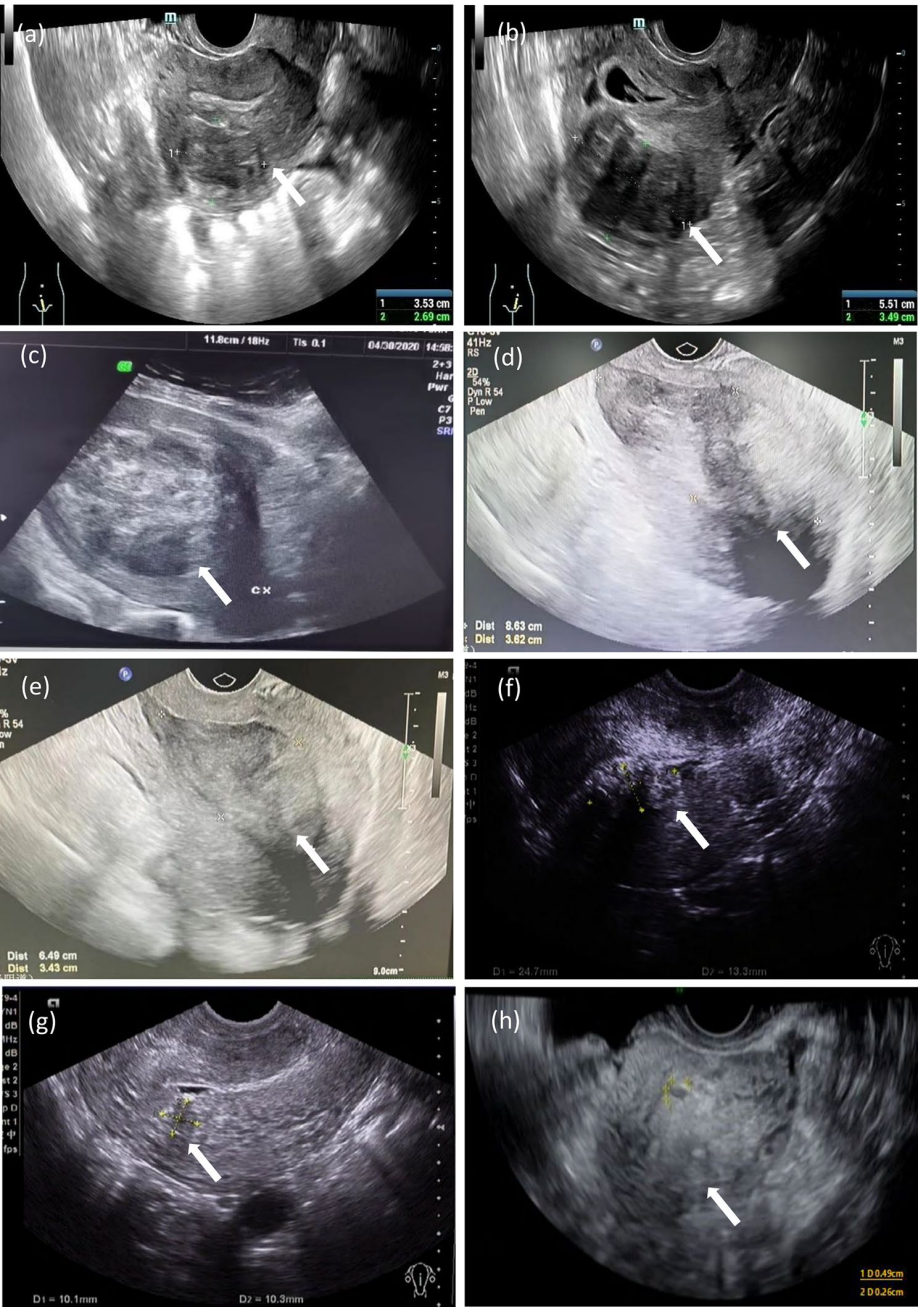
Ultrasound imgages from pre pregnancy to postpartum. (**a**) Ultrasound examination on December 14, 2018, showed multiple medium-to-low echogenicity (suggestive of leiomyomas) in the myometrium, with a larger one located in the posterior wall measuring approximately 3.5 × 4.0 × 2.7 cm (arrow). (**b**) Ultrasound examination on September 3, 2019, confirmed an early intrauterine pregnancy with an 8 mm embryo. Multiple medium-to-low echogenicity were observed in the myometrium, including a larger one in the posterior wall measuring approximately 5.5 × 3.5 cm (arrow), with a distinct boundary. (**c**) Ultrasound examination on April 30, 2020, 30 days postpartum, showed multiple heterogeneous hypoechoic masses in the uterine area. A large mass measuring 8.6 × 7.7 × 7.3 cm(arrow) was identified in the posterior wall. **(d)** Ultrasound examination on June 24, 2020 (> 2 months postpartum) showed an 8.6 × 3.6 cm hypoechoic mass (arrow) inside the uterine cavity with a distinct boundary. The hypoechoic mass inside the uterine cavity suggested a submucosal leiomyoma or a tissue residue. (**e)** Ultrasound examination on July 22, 2020, displayed a 6.5 × 3.4 cm hypoechoic mass (arrow) within the uterine cavity. **(f)** Ultrasound examination on April 19, 2021, indicated a 2.5 × 1.3 cm heterogeneous hypoechoic mass (arrow) within the uterine cavity. (**g**) Ultrasound examination on September 12, 2022, revealed a 1.0 × 1.0 cm hypoechoic mass (arrow) near the endometrium of the posterior wall of the uterine body. (**h**) Ultrasound examination on May 8, 2023, revealed no hypoechoic changes in the posterior wall (arrow)

The patient was a 40-year-old female, gravida 6, para 1, with a history of cesarean section. On December 14, 2018, an ultrasound examination (Fig. 1a) revealed multiple masses of medium-to-low echogenicity in the myometrium of the uterus, suggestive of leiomyomas. The largest leiomyoma in the posterior wall measured approximately 3.5 × 4.0 × 2.7 cm (Table 1). On September 3, 2019, the ultrasound results (Fig. 1b) confirmed an early intrauterine pregnancy. Multiple medium-to-low echogenicity lesions were observed in the myometrium, including a larger one in the posterior wall measuring approximately 5.5 × 3.5 cm. A cesarean section was performed on April 1, 2020 (36 + 4 weeks of gestation) due to premature rupture of membranes and a history of previous cesarean section. Intraoperatively, multiple leiomyoma-like masses were found in the posterior wall and bottom of the uterus, protruding into the serosal layer and not clearly into the mucosal layer. The most prominent lesion had a diameter of approximately 8.0 cm. After consulting with the patient, uterine myomectomy was withheld. One month after delivery (April 30, 2020), an ultrasound examination (Fig. 1c) showed multiple heterogeneous hypoechoic masses in the uterine region, with the largest one being 8.6 × 7.7 × 7.3 cm in the posterior wall, exhibiting capsular blood flow signals. Over two months after delivery, a follow-up ultrasound examination (Fig. 1d) revealed a hypoechoic mass of approximately 8.6 × 3.6 cm in the uterine cavity, displaying lacking blood flow signals. Myometrial echogenicity was homogeneous. No specific treatment was provided immediately, however, the patient reported lower abdominal distension and increased vaginal discharge. The leucorrhea test suggested vaginitis, and she was prescribed oral cefuroxime for 7 days. Over three months after delivery (July 24, 2020), a follow-up ultrasound examination showed a hypoechoic mass of approximately 6.5 × 3.4 cm in the uterine cavity, with visible blood flow signals and a resistance index (RI) of 0.44 (Table 1). The patient did not report significant abdominal pain, vaginal bleeding, or discharge during regular follow-up visits. The patient experienced a slightly increased menstrual flow as compared to before, without symptoms such as dizziness or weakness. Surgical treatment was recommended, but the patient declined. Annual follow-up ultrasound examinations (Fig. 1e, f and g) after delivery suggested a gradual reduction in the size of uterine leiomyomas. In the third postpartum year (May 8, 2023), the ultrasound examination (Fig. 1h) revealed no noticeable leiomyoma-like echoes in the posterior wall of the uterus (Table 1). During the whole treatment,the treatment principle is symptomatic management and regular follow-up under the condition of no severe complications, with surgical interventions pursued when required.

**Table 1:** Changes of uterine leiomyomas in the present case

Time	Stage	Site	Number of leiomyomas	Maximum size (cm)	Blood flow signal
2018-12-14	Pre-pregnancy	Myometrial	Multiple	3.5 × 4.0	
2019-09-03	Early pregnancy	Myometrial	Multiple	5.5 × 3.5	
2020-04-01	During cesarean section	Myometrial	Multiple	8.0–9.0	
2020-04-30	1 month after delivery	Myometrial	Multiple	8.6 × 7.7	Capsular blood flow signals
2020-06-24	> 2 months after delivery	Submucosal	Single	8.6 × 3.6	No blood flow signals
2020-07-22	> 3 months after delivery	Submucosal	Single	6.5 × 3.4	Visible blood flow signals, RI:0.44
2021-04-19	~ 1 year after delivery	Submucosal	Single	2.5 × 1.3	Visible blood flow signals, RI:0.49
2021-10-11	~ 1.5 years after delivery	Submucosal	Single	0.9 × 1.2	Few blood flow signals
2022-09-12	~ 2.5 years after delivery	Submucosal	Single	1.0 × 1.0	No significant blood flow signals
2023-05-08	> 3 years after delivery	No obvious leiomyoma-like echogenicity was found in the posterior wall of the uterine body.			

Note: RI, resistance index

## Discussion

Uterine leiomyomas are the most prevalent benign tumors affecting the female reproductive system, with an estimated incidence of pregnancy-associated uterine leiomyomas of approximately 10.7% [4]. During pregnancy, uterine leiomyomas may increase the risk of complications, such as miscarriage, preterm labor, and premature rupture of membranes. Uterine leiomyomas might lead to placental abruption during delivery, abnormal fetal positioning, obstructed labor, and postpartum hemorrhage. Furthermore, postpartum complications such as red degeneration of uterine leiomyomas might occur. In the reported case, the patient had multiple intramural uterine leiomyomas, including a giant uterine leiomyoma that did not protrude locally into the uterine cavity. These uterine leiomyomas did not significantly impact the pregnancy process from the early to late stages, and the delivery was successful. After delivery, the large intramural leiomyoma transformed into a submucosal leiomyoma, and all the submucosal and other multiple intramural uterine leiomyomas regressed and disappeared.

At the end of the pregnancy, the patient underwent a cesarean section due to a history of previous cesarean section and premature rupture of membranes. Intraoperatively, multiple leiomyomas were observed and the largest diameter was approximately 8.0 cm in the posterior wall of the myometrium, however, none protruded into the uterine mucosal layer. There is an ongoing debate regarding the performance of myomectomy during cesarean section. [5, 6]. Several studies suggest that simultaneous removal of uterine leiomyomas during cesarean section is relatively safe, such as small (< 6 cm) subserosal fibroids located on the anterior wall, with peduncles [7, 8]. However, other studies have identified risk factors for perioperative bleeding, including a leiomyoma measuring ≥ 7.0 cm in diameter, multiple leiomyomas, intramural [9, 10]. Moreover, women aged ≥ 40 years are at an increased risk of intraoperative bleeding [11]. Based on the above literature analysis and combined with the patient’s condition, no myomectomy was performed during the cesarean section.

The main concerns in the postpartum period for patients with uterine leiomyomas are susceptibility to red degeneration, late postpartum hemorrhage, and prolonged lochia. When the patient seeked medical attention for prolonged lochia, the ultrasound examination revealed a transformation of the intramural uterine leiomyoma into a submucosal one (Fig. 1d). Nkwabong et al. reported a case in which an intramural leiomyoma approximately 10.0 cm in diameter progressed into a submucosal leiomyoma during the puerperium and was eventually expelled from the vagina, later followed by a hysterectomy via the vaginal route [12]. Several reports had documented cases related to transformation of intramural leiomyomas into submucosal leiomyomas after UAE [13, 14]. The possible mechanism underlying the transformation is that large uterine leiomyomas are pushed toward the endometrial wall during uterine contractions. The intramural leiomyomas within the uterine wall could enter the uterine cavity through the uterine muscle layer between the endometrium and myometrium (which is susceptible to rupture due to ischemia or hypoxia in the endometrium and myometrium during postpartum or UAE). The patient was unwilling to undergo the operation and requested regular follow-up under the condition of no severe complications.After three years and one month, an ultrasound examination revealed a rare occurrence of the giant uterine leiomyoma regression.

Baird and Dunson [15] suggested that postpartum uterine involution induces leiomyoma regression. Uterine leiomyomas regression involves multiple mechanisms, including mechanical forces, vascular changes, hormonal changes, hypoxia, cellular apoptosis, and tissue development.

The shrinkage of postpartum uterine leiomyomas has been recognized to be associated with ischemia and hypoxia of uterine leiomyomas. The strong post-delivery uterine contractions promote remodeling of the uterus and uterine leiomyomas and cause blood vessel occlusion, resulting in ischemia and hypoxia in the myometrium and uterine leiomyomas. Uterine leiomyomas exhibit active growth and extensive cell division compared to typical uterine tissues, rendering them less tolerant to ischemia and hypoxia. The reperfusion of leiomyoma vessels is slower than the myometrium, leading to further ischemia, necrosis, and shrinkage of leiomyomas. Prior evidence has indicated that the mechanism of postpartum uterine leiomyoma regression is similar to UAE treatment for uterine leiomyomas [16].

Meanwhile, during uterine involution, the shrinking and degeneration of cells might secrete some growth-inhibitory factors, further promoting the regression of uterine leiomyomas. Uterine leiomyomas are also hormone-dependent tumors, and the withdrawal of hormones could induce apoptosis of hormone-dependent tissues [17]. However, hormone levels decrease significantly following delivery and expulsion of the placenta and fetus. This hormone shift promotes apoptosis, facilitating the further regression of postpartum uterine leiomyomas.

At over two months postpartum, the ultrasound examination of the patient revealed a transformation of the intramural uterine leiomyoma into a submucosal one. Laughlin et al. found a greater reduction in the diameter of submucosal uterine leiomyomas (1.8 cm) than in intramural leiomyomas (0.2 cm), subserosal leiomyomas (0.6 cm) or pedunculated leiomyomas (0.5 cm) [18]. In this case, the transformation of the intramural uterine leiomyoma into a submucosal leiomyoma further promoted the shrinkage of the uterine leiomyomas.

Currently, most studies indicate that postpartum regression of uterine leiomyomas is limited. A previous study has observed that the volumes of uterine leiomyomas decrease after 3 months postpartum compared to late pregnancy, with a 50% reduction of uterine leiomyomas between 3 and 6 months postpartum compared to early pregnancy [19]. However, approximately 10% of postpartum uterine leiomyomas may increase in size [20, 21]. In this case, the patient had multiple uterine leiomyomas before and during pregnancy, with one leiomyoma measuring 5.0 cm in early pregnancy and 8.0 cm in late pregnancy. Following postpartum uterine involution, the smaller uterine leiomyomas all regressed, and the large intramural leiomyoma transformed into a submucosal leiomyoma. After three years and one month postpartum, the ultrasound examination revealed complete regression of the large uterine leiomyoma. In this case, the submucosal leiomyoma further exacerbated ischemia and hypoxia, and facilitated the elimination of necrotic tissue from the fibroid. These factors may promote the complete regression of the large fibroid. Kim et al. published a case report of a patient who experienced complete regression of a large subserosa uterine leiomyomas four years after two deliveries [22]. It indicated that uterine leiomyomas may regress spontaneously after delivery and be needed longer observation time for postpartum uterine leiomyoma regression. More cases of postpartum uterine leiomyomas should be studied.

## Conclusions

Spontaneous regression of a giant uterine leiomyom is rare after delivery. The case report states that uterine leiomyomas may regress spontaneously after delivery and be needed longer observation time for postpartum uterine leiomyoma regression.The study and exploration of mechanisms involved in the regression of postpartum uterine leiomyomas will provide new insights for treatment options of uterine leiomyomas in the future.

## Data Availability

The datasets used and analyzed in this study are available from the corresponding author on reasonable request.
